# The Role of Regorafenib in the Management of Advanced Gastrointestinal Stromal Tumors: A Systematic Review

**DOI:** 10.7759/cureus.28665

**Published:** 2022-09-01

**Authors:** Vahe Khachatryan, Asmaa Muazzam, Chandani Hamal, Lakshmi Sai Deepak Reddy Velugoti, Godfrey Tabowei, Greeshma N Gaddipati, Maria Mukhtar, Mohammed J Alzubaidee, Raga Sruthi Dwarampudi, Sheena Mathew, Sumahitha Bichenapally, Lubna Mohammed

**Affiliations:** 1 Internal Medicine, California Institute of Behavioral Neurosciences & Psychology, Fairfield, USA; 2 Pathology, California Institute of Behavioral Neurosciences & Psychology, Fairfield, USA; 3 Research, California Institute of Behavioral Neurosciences & Psychology, Fairfield, USA

**Keywords:** treatment-related toxicity, survival rates, tyrosine kinase inhibitors (tki), metastatic gist, gastrointestinal stromal tumors (gists), regorafenib

## Abstract

Regorafenib, a multi-kinase inhibitor, has been widely used to treat patients with gastrointestinal stromal tumors (GIST) who failed the initial treatment with imatinib and sunitinib. This systematic review aims to demonstrate the efficacy and safety of regorafenib for patients with metastatic and/or unresectable GIST. We followed the Preferred Reporting Items for Systematic Reviews and Meta-Analyses (PRISMA) guidelines to perform this systematic review. We searched PubMed, Science Direct, and Cochrane databases to identify relevant articles based on predefined selection criteria. The implication of the search strategy results in 776 records from all databases. We excluded conference abstracts, discussion articles, case reports, case series, systematic reviews, and other observational non-intervention studies from the study, along with the articles published in languages other than English. After the screening and quality assessment, 10 studies were selected for final review - two randomized controlled trials and eight non-randomized prospective and retrospective review articles of intervention. Regorafenib improved the survival rates of patients after the failure of imatinib and sunitinib treatment, with an acceptable safety profile. Close monitoring of the patients may be needed to detect and manage the grade 4 or higher adverse events.

## Introduction and background

Gastrointestinal stromal tumors (GIST) are the most common tumors of mesenchymal origin arising in the gastrointestinal (GI) tract. Epidemiological studies showed a variable incidence of GISTs depending on geographical distribution, ranging from 4.3 per million to 22 per million new cases annually. The most common primary site of the tumor is the stomach (55.6%), followed by the small intestines (31.8%) and the colon (6%) [1]. The three main mutational subtypes of GISTs, accounting for about 95% of all tumor genotypes, are C-KIT-mutant (receptor tyrosine kinase protein known as tyrosine-protein kinase KIT), which is also known as cluster of differentiation 117 (CD117)-mutant, platelet-derived growth factor receptor alpha (PDGFRA) -mutant, and succinate dehydrogenase (SDH) -deficient. Approximately 70% of all mutations are due to activating mutations of exon 11 (60%) and exon 9 (9-10%) of the C-KIT gene. PDGFRA mutations account for 15%, and about 9% are SDH-deficient subtypes [2]. Small subsets of GISTs have activating mutations in the BRAF gene, also known as proto-oncogene B-Raf (V600E mutation), and loss-of-function mutations in the neurofibromin (NF-1) gene. These tumors are known as wild-type GISTs [3].

The first-line treatment for metastatic and/or unresectable advanced GISTs is imatinib mesylate (IM). IM is a protein-tyrosine kinase inhibitor (TKI) that competitively inhibits C-KIT (CD117), PDGFRA, and other tyrosine kinases. It has been approved for the treatment of advanced GIST with a recommended dose of 400mg/day taken by mouth for up to three years [4]. As a first-line agent, imatinib considerably impacts the survival rates of advanced GISTs and remains crucial for managing these patients [5]. However, about 10%-15% of patients develop resistance to IM within the first six months of the treatment [6,7].

Most primary TKI resistance is due to a D842V mutation in the PDGFRA gene. A study of patients with this mutation treated with TKIs showed poor outcomes with the lowest rates of survival and no clinical benefit [8]. The other subgroup of patients with primary resistance was shown to lack any mutations in C-KIT and PDGFRA genes, most of which demonstrated mutations in the succinate dehydrogenase X (SDHX) gene [9]. Secondary mutations tend to occur in the same gene and loci as the primary gene mutation. Exon 11 of the C-KIT gene is the most commonly reported site of secondary mutation in tumors with preexisting primary mutation [10].

An ongoing trial assessing the alternating regimen of imatinib with regorafenib versus imatinib alone for the initial management of advanced GIST evaluates the possibility of overcoming or delaying the secondary TKI resistance problem. Early analysis of data showed no substantial difference in objective tumor response and progression-free survival rates between the experimental groups [11].

Imatinib-resistant patients can be treated with the second-line multi-kinase inhibitor sunitinib malate (SM) with a recommended dose of 50mg once daily in six-week cycles with four weeks on medication and two weeks off until progression of the disease or unmanageable toxicity [12]. INTRIGUE study reported similar benefits of ripretinib as a second-line therapy, compared to sunitinib, with a more tolerable safety profile and patient-reported outcomes (PRO) [13].

**Figure 1:**
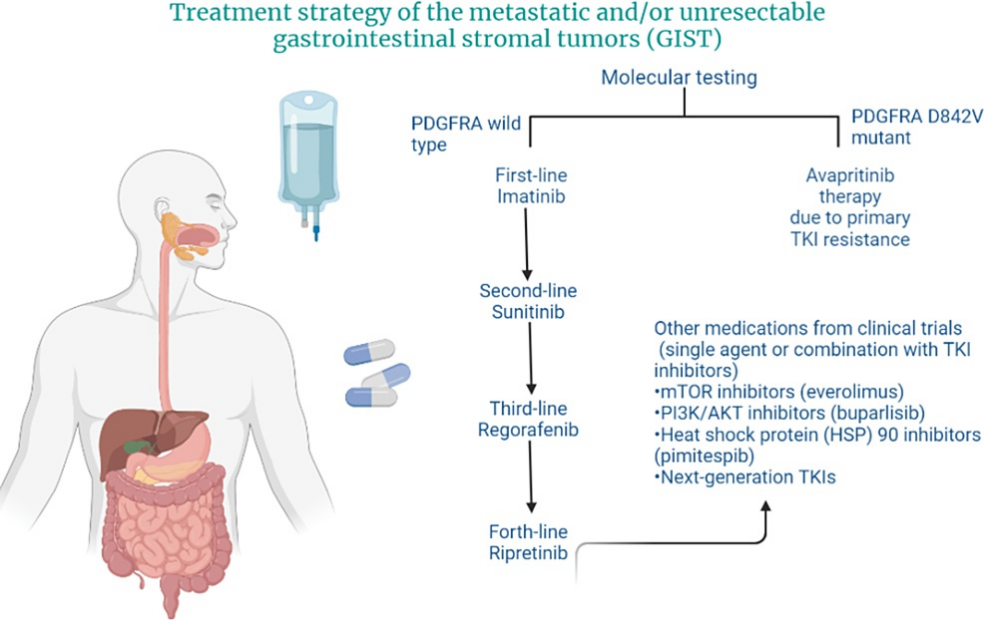
Treatment of advanced gastrointestinal stromal tumors (GIST) PDGFRA: Platelet-derived growth factor receptor alpha; TKI: Tyrosine-kinase inhibitor; mTOR: Mammalian target of rapamycin; PI3K-AKT:  Phosphatidylinositol-3-Kinase and Protein Kinase B Created by the author using BioRender.com

After both imatinib and sunitinib fail, the other multi-kinase inhibitor regorafenib is indicated as a third-line treatment option. It targets different angiogenic (vascular endothelial growth factor receptor (VEGFR)-1, -2, and -3, and angiopoietin-1 receptor (TIE2)), oncogenic (KIT, Ret protooncogene, RAF-1 protooncogene, BRAF, and BRAFV600E), and stromal (PDGFR and fibroblast growth factor receptor (FGFR)) TKs with noticeable survival benefits in patients with advanced GIST resistant to IM and SM [14]. Multiple studies showed the efficacy of regorafenib for metastatic colorectal cancer, hepatocellular carcinoma, and advanced GIST [15-17]. There is promising data about regorafenib activity in other tumors, such as gastroesophageal cancer, cholangiocarcinoma, glioblastoma, and sarcomas other than GIST [18]. Apart from IM, SM, and regorafenib, the Food and Drug Administration (FDA) has already approved newer drugs, including ripretinib and avapritinib, for the treatment of advanced GISTs resistant to third-line therapy [19,20]. Figure 1, illustrated by the authors, summarizes the treatment of advanced GIST.

Although many studies showed the efficacy of regorafenib for managing metastatic and/or unresectable, advanced GIST resistant to first-line and second-line treatments, there is no definitive conclusion to prove the effectiveness and the safety of this medication. Therefore, this systematic review aims to show regorafenib's efficacy and safety for treating metastatic and/or unresectable GIST in patients after a failure of imatinib and sunitinib.

## Review

Methods

We used the Preferred Reporting Items for Systematic Reviews and Meta-Analyses (PRISMA) 2020 guidelines to conduct this study [21].

Eligibility Criteria

We identified specific inclusion criteria to select the studies relevant to our systematic review. Studies evaluating the role of regorafenib in metastatic and/or unresectable advanced gastrointestinal tumors after the failure of imatinib and sunitinib were selected as the primary target of our research. We selected randomized and non-randomized studies and intervention review articles from different medical centers for a systematic review. We defined the study participants' age to be 18 years and above. Articles published in languages other than English were excluded, along with conference abstracts, discussion articles, case reports, case series, systematic reviews, and other observational non-intervention studies.

Databases and Search Strategy

We systematically searched the PubMed, ScienceDirect, and Cochrane Library databases to identify the relevant articles and include them in our systematic review. The last date for the search was May 17, 2022. We used keywords and Medical Subject Headings (MeSH) terms to create the search strategy for all databases, as shown in Table 1.

**Table 1:** Search strategies and the number of results identified from systematic search. GIST: Gastrointestinal stromal tumors; MeSH: Medical Subject Heading; tiab: Title and abstract; majr: Major topic

Databases	Keywords	Search strategy	Filters	Results
PubMed	Gastrointestinal stromal tumors; GIST; regorafenib; advanced gastrointestinal stromal tumors; Metastatic and/or unresectable GIST	#1 'Gastrointestinal stromal tumors'[Mesh] OR 'gastrointestinal stromal tumor'[tiab] OR 'gastrointestinal stromal tumors'[tiab] OR 'gastrointestinal stromal tumor'[majr] OR 'GIST'[tiab] OR 'GIST'[majr] #2 'regorafenib'[tiab] OR 'regorafenib'[majr] OR "regorafenib" [Supplementary Concept] #3 'metastatic'[tiab] OR 'metastatic'[majr] OR 'advanced'[tiab] OR 'advanced'[majr] OR "Neoplasm Metastasis"[Mesh] #4 ('Gastrointestinal stromal tumors'[Mesh] OR 'gastrointestinal stromal tumor'[tiab] OR 'gastrointestinal stromal tumors'[tiab] OR 'gastrointestinal stromal tumor'[majr] OR 'GIST'[tiab] OR 'GIST'[majr] OR 'gastrointestinal stromal tumour'[tiab] OR gastrointestinal stromal tumors OR GIST) AND ('regorafenib'[tiab] OR 'regorafenib'[majr] OR regorafenib OR "regorafenib" [Supplementary Concept]) AND ('metastatic'[tiab] OR 'metastatic'[majr] OR 'advanced'[tiab] OR 'advanced'[majr] OR "Neoplasm Metastasis"[Mesh] OR metastatic OR advanced)	English	177
ScienceDirect	Gastrointestinal stromal tumors, regorafenib, advanced GIST	Gastrointestinal stromal tumors and regorafenib GIST and regorafenib	Research articles; Review Articles	518
Cochrane Library	Gastrointestinal stromal tumors, regorafenib, advanced GIST	Gastrointestinal stromal tumours and regorafenib	-	81 trials

The references found in the databases were organized and alphabetized by Microsoft Excel (Microsoft Corporation, Redmond, Washington, United States), and duplicate articles were removed. We initially screened the articles by titles and abstracts to rule out irrelevant articles. Subsequently, we conducted a full-text review of the remaining manuscripts to select the studies for further quality assessment.

Risk of Bias Assessment

All selected articles were evaluated for the risk of bias and quality assessment using tools based on the study designs: Cochrane Collaboration Risk of Bias Tool (CCRBT) for randomized controlled trials (RCTs) and Risk of Bias In Non-randomised Studies - of Interventions (ROBINS-I) tool for non-randomized trials (N-RCT) and review articles [22,23]. We evaluated each study using specific domains for the quality assessment and defined each domain as having low risk, moderate risk, high risk, and no information. If at least 70% of the domains were low risk, the study was selected for systematic review.

Data Collection and Analysis

We developed a standardized template using Microsoft Excel (Microsoft Corporation, Redmond, Washington, United States) to extract the following data from the selected studies: first author name, publication year, recruitment time, sample size, follow-up duration, treatment regimen, reported adverse events, and outcomes. We performed a descriptive analysis of data to show the efficacy of regorafenib in advanced GIST after the initial failure of imatinib and sunitinib.

Outcome Measures

The primary outcome of our systematic review was the evaluation of the efficacy and safety profile of regorafenib in advanced GIST. The outcome measures for this systematic review were survival rates (overall survival (OS), event-free survival (EFS), progression-free survival (PFS)) as well as response measures, including complete response (CR), partial response (PR), and stable disease (SD) rate if applicable.

Results

Literature Search and Selection of Studies

**Figure 2:**
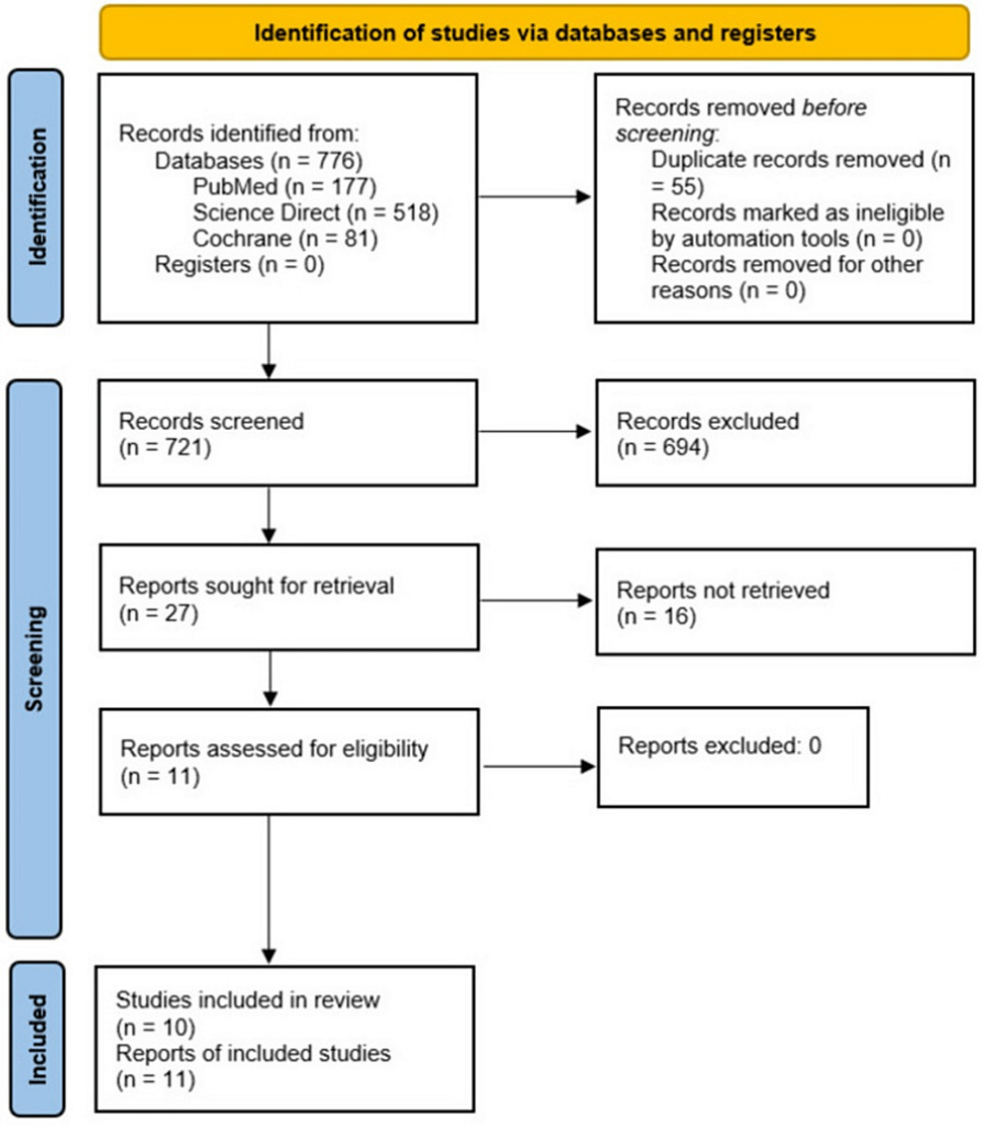
PRISMA flow diagram of the systematic review. PRISMA: Preferred Reporting Items for Systematic Reviews and Meta-Analyses

We searched three databases (PubMed, ScienceDirect, Cochrane) for all potentially relevant articles for this systematic review. The implication of a predetermined search strategy revealed 776 records. After removing duplicates, all remaining records were initially screened by reading the title and abstract. A secondary review was done by reading the full-text manuscripts to rule out irrelevant articles and studies lacking the required data for this systematic review. We created a PRISMA flowchart of study identification shown in Figure 2 [21].

Risk of Bias

After selecting relevant articles, we evaluated the quality of included manuscripts to assess the risk of bias. Tables 2, 3 summarize the quality assessment with two different tools.

**Table 2:** Risk of bias assessment in randomized controlled trials. CCRBT: Cochrane Collaboration Risk of Bias Tool

Cochrane's tool CCRBT	Random sequence generation	Allocation concealment	Blinding of participants and researchers	Blinding of outcome assessment	Incomplete outcome data	Selective reporting	Other bias
Demetri et al. 2013 [17]	Low Risk	Low risk	Low risk	Low Risk	Low Risk	Low Risk	Low Risk
Kang et al. 2021 [24]	Low Risk	Low Risk	High Risk	Moderate Risk	Low Risk	Low Risk	Low Risk

**Table 3:** Risk of bias assessment in prospective and retrospective reviews of intervention. ROBINS-I: Risk of Bias In Non-randomised Studies - of Interventions

ROBINS-I (Non-randomized trials and reviews)	Bias due to confounding	Bias in selection of participants into the study	Bias in classification of interventions	Bias due to deviations from intended interventions	Bias due to missing data	Bias in measurement of outcomes	Bias in selection of the reported result
Ben-Ami et al. 2016 [25]	No Information	Low Risk	Low Risk	High Risk	Low Risk	Low Risk	Low Risk
George et al. 2012 [26]	No Information	Low Risk	Low Risk	High Risk	Low Risk	Low Risk	Low Risk
Hu et al. 2020 [27]	No Information	Low Risk	Low Risk	Moderate Risk	Low Risk	Low Risk	Low Risk
Kim et al. 2019 [28]	No Information	Moderate Risk	Low Risk	Low Risk	Low Risk	Low Risk	Low Risk
Kollar et al. 2014 [29]	No Information	High Risk	Low Risk	Low Risk	Low Risk	Low Risk	Low Risk
Nannini et al. 2021 [30]	No Information	Low Risk	Moderate Risk	Low Risk	Low Risk	Low Risk	Low Risk
Son et al. 2017 [31]	No Information	Low Risk	Low Risk	Low Risk	Low Risk	Low Risk	Low Risk
Yeh et al. 2017 [32]	No Information	High Risk	Low Risk	Low Risk	Low Risk	Low Risk	Low Risk
Teranishi et al. 2022 [33]	No information	Low Risk	Low Risk	Low Risk	Low Risk	Low Risk	Low Risk

Study Characteristics

This systematic review revealed two RCTs and eight prospective and retrospective studies of interventions showing the efficacy and safety of regorafenib in treating metastatic and/or unresectable advanced gastrointestinal stromal tumors after the failure of imatinib and sunitinib regimens. Demetri et al. (2013) conducted a phase III randomized controlled trial which became the cornerstone for the approval of the drug by the FDA [17].

The standard treatment regimen in all studies was the same: regorafenib 160 mg/daily in four-week cycles with three weeks on medication and one week off of it. Nannini et al. (2021) assessed the effect of personalized dose reductions and temporary interruptions to control and prevent drug-related adverse events [30]. The second phase III RCT performed by Kang et al. (2021) evaluated the avapritinib vs. regorafenib for patients with locally advanced, unresectable, and/or metastatic GIST previously treated with imatinib and one or two other TKIs (excluding avapritinib and regorafenib) [24]. Data from all selected studies are demonstrated in Table 4.

**Table 4:** Data and characteristics of individual studies. N/A: Non-applicable; R-ST: Regorafenib standard treatment (160mg daily 28-day cycles, 21 days on medication, seven days off drug); Avap: Avapritinib (300mg daily 28-day cycles); PFS: Progression-free survival, OS: Overall survival, CR: Complete response, PR: Partial response, SD: Stable disease, PD: Progressive disease, HTN: Hypertension, HFS: Hand-foot-skin reaction

First author	Study phase	Recruitment	Sample size	Follow-up	Treatment	Survival rates	Response rates	Adverse events
George et al. 2012 [26]	II	Feb 2010- Dec 2010	33	10.9 months	R-ST	PFS - 10 months	CR+PR+SD-75% of 16+weeks; (SD-63.4%, PR- 11.6%)	HTN-36%; HFS-24%, hypophosphatemia-15%
Demetri et al. 2013 [17]	III	Jan 2011- Aug 2011	133	22.9 months	R-ST: placebo 2:1	PFS-4.8 months	PR- 4.5%, SD-71.4%	HTN-23.5%, HFS-19.7%, Diarrhea-5.3%
Kollar et al. 2014 [29]	N/A	March 2013 - Sept 2013	20	12.6 months	R-ST	PFS-9.4 months, OS-12.2months	PR-11%, SD-11%,	Fatigue-80%, HFS-55%, HTN-50%, diarrhea-50%
Ben-Ami et al. 2016 [25]	II	Feb 2010- Jan 2014	33	41 months	R-ST	PFS-13.2 months; OS-25months	PR-18.2%, SD-57.6%	HFS-91%, fatigue-85%, diarrhea-79%, HTN-76%
Son et al. 2017 [31]	N/A	Dec 2012-Nov 2013	57	12.7 months	R-ST	PFS-4.5 months, OS-12.9 months	SD-44% (>12 weeks)	HFS-25%, HTN-7%, rash-7%
Yeh et al. 2017 [32]	II	June 2014- May 2016	18	10.9 months	R-ST	PFS-22.1 months	PR-40%, SD-53.3%, PD-6.7%	HFS-55.6%, HTN-27.8%, hepatotoxicity-16.7%, fatigue-5.6%
Kim et al. 2019 [28]	II	Sep 2016- Aug 2017	25	8.6 months	Continuous dose regorafenib, 100mg daily for 28 days	PFS-7.3 months	PR-8%, SD-56%, PD-32%	HFS-16%, hepatotoxicity-8%
Hu et al. 2020 [27]	N/A	April 2014- Dec 2017	28	14.8 months	R-ST	PFS-4.4 months, OS-29.3 months	PR-14.3%, SD-35.7%, PD-35.7%	HTN-71.4%, anemia-67.8%, HFS-64.3%
Nannini et al. 2021 [30]	N/A	Feb 2013 - Jan 2021	152	36.5 months	114 (75%) R-ST:38 (25%) adjusted dose	PFS 5.6 months (Standard), 9.7 months (Personalized);	N/A	N/A
Kang et al. 2021 [24]	III	March 2018 - Nov 2019	236	9.6 months	Avap: R-ST	mPFS 5.6 months	SD-67.4%, PD-20.8%	Fatigue-34.2%, diarrhea-34.6%, HFS-59%
Teranishi et al. 2022 [33]	N/A	Aug 2013 - Sep 2021	33	20.6 months	R-ST	OS 23.8 months, PFS 7.1 months	PR-9.1%, SD-51.5%, PD-30.3%	HFS-72.7%, hepatotoxicity-33.3%, diarrhea-27.3%

Survival Rates

In phase III randomized controlled trial conducted by Demetri et al. (2013), the median PFS was 4.8 months compared to the placebo group, where it was 0.9 months (Hazard ratio (HR): 0.27, 95% confidence interval (CI) 0.19-0.39; p<0.0001). There was no statistically significant difference in OS between the two groups (HR: 0.77, 95% CI 0.42-1.41; p=0.199) [17]. The second RCT evaluated the avapritinib vs. regorafenib for the target population and showed no significant difference in median PFS between the two medication groups (HR: 1.25; 95% CI, 0.99 to 1.57; mPFS 4.2 vs. 5.6 months, respectively; P=0.055) [24].

George et al. (2013) showed 10 months of median PFS (95% CI, 8.3 to 14.9 months) in a multicenter Phase II trial [26]. Long-term follow-up of this study showed a median OS of 25 months (95% CI 13.2-39.1 months), one-year OS rate of 79% (95% CI 61% to 89%), and mPFS of 13.2 months (95% CI 9.2-18.3 months) [25]. A report from the UK study conducted by Kollar et al. (2014) demonstrated an mPFS of 9.4 months (95% Cl: 6.2-not calculable) and six-month PFS of 80.0% (95% CI 55.1-92.0), along with mOS and six-month OS of 12.2 months (95% Cl: 10.5-not calculable) and 89.7% (95% CI 64.8-97.3), respectively [29]. A prospective study of 57 patients from three medical institutions in Korea revealed 4.5 months of median PFS (95% CI, 3.8 to 5.3) and 12.9 months of median OS (95% CI, 8.1 to 17.7) [31]. Yeh et al. (2017) showed significant improvement in median PFS with an intervention group compared to a historical cohort (22.1 vs. 5.5 months, p = 0.0001) [32]. Kim et al. (2019) illustrated the effect of continuous dosing of regorafenib for advanced GIST patients and showed mPFS of 7.3 months (95% CI, 5.9-8.6 months) and a one-year survival rate of 64.5% [28]. A prospective Taiwanese study of 28 patients revealed an mPFS of 4.44 months and an OS of 29.34 months [27]. Nannini et al. (2021) compared the standard versus personalized dosing regimens for advanced GIST in a retrospective multicenter study, resulting in mPFS of 5.6 months (95% CI 3.73-11.0 months) versus 9.7 months (95% CI 7.9-14.5 months), respectively (HR 0.51; 95% CI 0.34-0.75; P = 0.00052), and a mOS of 16.6 months (95% CI 14.1-21.8 months) versus 20.5 months (95% CI 15.0-25.4 months), respectively (HR 0.75; 95% CI 0.49-1.22; P = 0.16) [30]. The latest Japanese study by Teranishi et al. (2022) demonstrated 23.8 months of median OS and an 80% rate of one-year OS, along with 7.1 months of mPFS and a 40.2% rate of one-year PFS [33].

Response Rates

Clinical response to the treatment was assessed by response evaluation criteria in solid tumors (RECIST) 1.1 criteria, including complete response (CR), partial response (PR), stable disease (SD), and progressive disease (PD) [34]. Demetri et al. (2013) reported PR of 4.5%, SD of 71.4%, and disease control rate (DCR) of 52.6% [17]. Kang et al. (2021) showed 46.5% DCR (95% CI, 39.9 to 53.2), and SD and PD were 67.4% and 20.8%, respectively [24].

There was a 75% clinical benefit (CR+PR+SD) (95% CI: 61%-91%) in a multicenter Phase II trial by George et al. (2013), with an SD of 63.4% and PR of 11.6% [26]. Ben-Ami et al. (2016) showed a PR of 18.2% and an SD of 57.6% [25]. A UK study by Kollar et al. (2014) demonstrated 11% PR and 11% SD [29]. Son et al. (2017) reported 44% SD after 12 weeks of treatment [31]. Yeh et al. (2017) showed promising results with PR of 40%, SD of 53.3%, and PD of 6.7% [32]. Patients in the study conducted by Kim et al. (2019) had PR of 8%, SD of 56%, and PD of 32% [28]. A Taiwanese study by Hu et al. (2020) reported 14.3% PR, 35.7% SD, and 35.7% PD [27]. Teranishi et al. (2022) demonstrated PR of 9.1%, SD of 51.5%, and PD of 30.3% in a study of Japanese patients [33].

Discussion

This study aims to demonstrate the efficacy and safety of regorafenib in patients with advanced metastatic and/or unresectable gastrointestinal tumors after the failure of initial imatinib and sunitinib therapy. We will discuss the survival rates and treatment-related adverse events of regorafenib in selected studies and mention the limitations of our systematic review to highlight potential bias associated with the data.

Randomized Controlled Trials

We identified two randomized controlled trials, one of which compared regorafenib to placebo in a target population. Demetri et al. proved the superiority of regorafenib over placebo in a phase III GRID study of 199 patients. Although there was no complete response to the regorafenib treatment, they showed a clinically significant tumor control rate with a disease control rate (DCR) of 52.6% compared to 9.1% in the placebo group [17]. Subgroup analysis of Japanese patients from the GRID trial showed survival rates similar to the overall study population and a slightly higher stable disease (SD) rate of 92% compared to 71% in a general study group [35].

Kang et al. investigated the difference between outcomes in patients treated with avapritinib versus regorafenib [24]. There were no statistically significant benefits for the avapritinib compared to regorafenib in imatinib-resistant individuals as a third-line or later treatment for advanced GIST.

Non-Randomized Studies

Phase II prospective study showed statistically significant improvement in clinical benefit rates (CBR), the most prominent in patients with C-KIT exon 11 mutations and SDH-deficient subtypes. There is an 83% metabolic response in patients with exon 11 mutations [25]. Conversely, in a Korean study of 57 patients, there is no statistically significant difference in survival rates between patients with primary KIT exon 11, exon 7 mutations, or wild-type GISTs [31]. Regorafenib may also be beneficial for patients with exon 17 mutations of the KIT gene, as Yeh et al. showed promising results of a 93.3% disease control rate after 16 weeks on medication [32]. Continuous lower dose treatment without an off period was comparable to the standard regimen with a similar tumor response rate but more manageable adverse events [28].

The survival rates reported in a prospective single-center study of Taiwanese patients showed similarity with the results of the other studies on the Asian population, including Korean and Japanese patients [27,31,35].

Overall, survival rates in the randomized studies were comparable. However, non-randomized studies showed higher than expected survival rates, probably because of confounding factors, such as ethnicity, follow-up duration, age of the participants, or study designs. On the other hand, response rates reported in selected studies were diverse, mainly because of the different follow-up periods.

Toxicity

One of the main reasons for treatment discontinuation in studies of regorafenib is treatment-related toxicities. The most common adverse event (AE) noted in regorafenib studies was palmar-plantar erythrodysesthesia (PPE), more commonly known as hand-foot skin (HFS) reaction comprising 16%-91% of reported grade 3 and more adverse events in studies included in our systematic review, the lowest noted in a study of continuous low-dose regorafenib treatment [28]. A meta-analysis of the incidence of HFS in randomized controlled trials of patients treated with regorafenib showed 54% of all-grade HFS and 16% of high-grade HFS, with a bit of variation depending on the tumor type [36]. PPE starts with a prodromal stage when patients notice a tingling or burning sensation lasting for a few days, followed by tender, bilateral erythema of affected skin with a formation of blisters. Studies suggest using supportive measures when this reaction occurs. Topical emollients, analgesics, or, in more advanced cases, topical steroids and keratolytic agents can be used cautiously and limited only to the affected parts of the skin [37,38]. The second most common AE was hypertension (7%-76%), a high-risk adverse event that needs careful management and control. Regorafenib-induced increase in blood pressure (BP) is usually transient and returns to its initial state with discontinuation of the medication. Grade 2 (BP more than 140/90 mmHg) and more hypertension should be managed with the addition of antihypertensive drugs, starting with angiotensin-converting enzyme (ACE) inhibitors. Beta-blockers and calcium-channel inhibitors may be added in case of failure to control BP [39]. Other adverse events noted in selected studies were fatigue and diarrhea, which impact patients’ quality of life and activities of daily living and should be managed conservatively. Studies suggest monitoring liver function tests biweekly because of the liver toxicity associated with regorafenib. Management is usually limited to dose adjustments, temporary interruptions, or medication discontinuation [12,39].

Overall, regorafenib has a sustainable toxicity profile which can be managed by decreasing the dosage, supportive measures, or temporary interruptions in the treatment.

Limitations

This systematic review has some limitations that are worth mentioning. We excluded articles published in languages other than English in this study. We performed a descriptive analysis of data from included studies. Therefore, there is a need for a comprehensive meta-analysis of survival rates and toxicity. The small number of randomized controlled trials evaluating the efficacy of regorafenib and the small sample size in selected studies are the most important limitations of our systematic review.

## Conclusions

In conclusion, studies included in our systematic review demonstrated a beneficial impact of regorafenib on survival rates of patients with advanced gastrointestinal stromal tumors after the failure of imatinib and sunitinib treatment. Dose adjustments can be crucial during the therapy to improve the tolerability of the medication. The toxicity profile of the regorafenib is manageable, with hand-foot skin reaction and hypertension being most commonly seen. Educating patients about the possibilities of these reactions and the need to seek medical attention when they appear can favor the continuity of the treatment. We also recommend performing molecular testing of tumors to identify specific mutations that can impact patients' future management.

There is an implication for future research to conduct randomized controlled clinical trials comparing regorafenib with other medications to demonstrate the superiority of the drug in treating advanced gastrointestinal stromal tumors. The issue of drug resistance persists, so future studies should investigate the detailed mechanisms of primary and secondary resistance and conduct trials with newly developed medications to overcome the problem of TKI resistance.
